# Exploring Neighborhood Opportunity as a Factor in Pediatric Asthma Visits to the Emergency Department

**DOI:** 10.1177/00333549251361324

**Published:** 2025-08-31

**Authors:** Nima Khodakarami, Marvellous Akinlotan, Alva O. Ferdinand

**Affiliations:** 1 Department of Health Policy and Administration, Penn State University, Monaca, PA, USA; 2 Center for Applied Studies in Health Economics, College of Medicine, Penn State University, Hershey, PA, USA; 3 Southwest Rural Health Research Center, School of Public Health, Texas A&M University, College Station, TX, USA; 4 Texas A&M College of Dentistry, Dallas, TX, USA; 5 Department of Health Policy and Management, Texas A&M University, College Station, TX, USA

**Keywords:** neighborhood effects, asthma, emergency department, children, health disparity

## Abstract

**Objectives:**

Despite growing interest in environmental and social determinants of health, few studies have explored how residential mobility influences respiratory health outcomes. We examined the relationship between levels of opportunity across education, health and environment, social and economic, and all domains in a child’s neighborhood and the likelihood of emergency department (ED) visits for asthma and showed how moving from one neighborhood to another would affect the odds of visiting the ED for asthma.

**Methods:**

In this cross-sectional study, we analyzed asthma-related ED visits among children aged 2 to 17 years in 9 US states (Arizona, Florida, Kentucky, Maryland, New Jersey, North Carolina, Oregon, Rhode Island, and Wisconsin) during 2016-2019. We used a multivariable logistic regression model to examine the relationship between the Child Opportunity Index (COI) and ED visits for asthma. We used a piecewise linear logit model to estimate the neighborhood’s opportunity effect.

**Results:**

Children living in neighborhoods with very low COI had a high probability of visiting the ED for asthma (adjusted odd ratio = 1.14; *P* < .001). In addition, moving from a low to a very low COI neighborhood significantly increased the probability of asthma-related ED visits among children aged 5 to 9 years (0.8 percentage points), Black children (0.4 percentage points), boys (0.7 percentage points), and those living in large metropolitan areas (0.6 percentage points).

**Conclusions:**

Our findings suggest that improvement in neighborhood opportunity may translate to better asthma-related health outcomes among children. Future research should continue to investigate the effects of neighborhood opportunity on other childhood conditions.

In the United States, emergency department (ED) visits are the most common way children receive care for asthma, accounting for half a million visits annually.^1-3^ While these visits are costly, averaging $624 per child in 2024 US dollars,^4^ nearly 30% are potentially preventable.^5,6^ As a result, reducing ED visits for asthma by addressing factors such as social determinants of health (SDOH) that contribute to a child’s health outcomes has become a key objective of the Healthy People 2030 initiative.^7^

Previous studies examined the unidimensional association between pediatric ED visits for asthma and SDOH factors, primarily focusing on the isolated effects of health insurance coverage, demographic characteristics, or median annual household income.^8-11^ New research, however, is increasingly directed toward describing the entwined effects of SDOH by using the context of a neighborhood where children, live, play, and grow.^11-14^ For example, one study that examined the effect of a child’s neighborhood opportunity on ED visits for asthma in Florida found that children who lived in tracts with high neighborhood opportunity generally had fewer asthma-related ED visits than did children who lived in tracts with low neighborhood opportunity.^15^ Another study that investigated the relationship between a child’s neighborhood opportunity and the diagnoses of pediatric ED visits for asthma in San Francisco found, after adjusting for neighborhood income and patient characteristics, reduced odds for asthma-related ED visits in neighborhoods with high opportunity.^16^ A study in Illinois, which evaluated the percentage of pediatric ED encounters by a child’s neighborhood opportunity, showed that the distribution of asthma diagnoses was high in areas with lower levels of neighborhood opportunity.^17^ Additional studies have shown that the probability of children repeatedly going to the ED for asthma was high among those with lower neighborhood opportunity levels.^18,19^

In this study, we examined the odds of ED visits for asthma across neighborhoods with various levels of resources. We used the Child Opportunity Index (COI) to measure the neighborhood effect on pediatric ED visits for asthma.^20,21^ Although several studies examined the relationship between the COI metric and ED visits, they were limited by a small number of hospitals or a focus on inpatient use.^22-26^ Our study complements previous research in that it uses a large sample of data to estimate how moving from one neighborhood to another would affect the odds of visiting the ED for asthma. We further examined the influence of race and ethnicity at all levels of COI, which, to our knowledge, has been examined only in a study that focused on patients with diabetes.^27^ We focused on the health and environmental opportunity dimensions of a neighborhood. The large size of our data enabled us to explore possible mechanisms of the effects of neighborhood opportunity levels on the probability of visiting the ED for asthma. Furthermore, the richness of individual characteristics made it possible to examine neighborhood effects on less investigated groups, such as those living in nonmetropolitan/noncore areas that are not adjacent to metropolitan or micropolitan areas and do not have their own town.

## Methods

### Study Data

In this retrospective observational study, we examined ED visits for asthma among children aged 2 to 17 years. To obtain information on children with asthma, we used data from the State Emergency Department Databases (SEDD), which are made available by the Healthcare Cost and Utilization Project (HCUP) at the Agency for Healthcare Research and Quality (AHRQ) for the years 2016 through 2019.^28^ We selected the states included in this study (Arizona, Florida, Kentucky, Maryland, New Jersey, North Carolina, Oregon, Rhode Island, and Wisconsin) based on data availability. We further restricted our sample to ED visits in which the patient’s county of residence was congruent with the state in which ED services were obtained.

To capture the complex contexts of neighborhoods, we used the publicly available COI 2.0 data.^20^ The COI 2.0 metric categorizes neighborhoods into 5 opportunity levels that indicate the availability of resources that are essential for children’s healthy development: very low, low, moderate, high, and very high. Domains are specified for education, health and environment, social and economic factors, and an overall metric combining all 3 domains. The individual indicators are weighted according to their effect on child health and well-being and comprise multiple factors. The education domain captures early childhood, elementary, secondary, and postsecondary education as well as educational and social resources. The health and environment domain characterizes neighborhoods by the extent of their healthy environments, toxic exposures, and health resources. The social and economic domain includes economic opportunities as well as economic and social resources.^21,29,30^

### Outcome Measures

The outcome was ED visits for asthma. We identified the outcome using the AHRQ Pediatric Quality Indicator 14 (PDI 14) as used in previous studies.^31^ We used *International Classification of Diseases, 10th Revision, Clinical Modification* (ICD-10-CM) codes to select cases with a principal code of J45 asthma, which includes 13 ICD-10-CM codes indicating a range of mild to severe or unspecified intermittent and persistent asthma with acute exacerbation and other asthma types.^32^ Consistent with the AHRQ algorithm, we also excluded cases that were coded as involving cystic fibrosis or respiratory system anomalies; transfer from another facility; specific point-of-origin codes; major diagnostic category (MDC 14) for pregnancy, childbirth, and the puerperium; and missing data on sex, age, quarter, year, principal diagnosis, or county.^31^

### Exposure Variable

We used neighborhood opportunity indicators that classify neighborhoods on a scale of 1 to 5, where 1 = very low opportunity and 5 = very high opportunity. We used COI scores as our exposures. In all analyses, neighborhoods with very high opportunity levels were the reference group.

### Covariates

We controlled for the characteristics of patients, which were available for each discharge record in the studied states. We grouped patient age into 2 to 4, 5 to 9, 10 to 14, and 15 to 17 years to be consistent with the AHRQ 2020 population file age grouping. Other patient characteristics included sex, race and ethnicity (White, Black, Hispanic, and other race), and urban–rural designation for the patient’s county of residence (large metropolitan, small metropolitan, micropolitan, and nonurban residence).

### Analyses

We used a multivariable logistic regression model to examine the relationship between neighborhood opportunity levels and ED visits for asthma. The model controlled for state and year fixed effects and various individual characteristics (eg, age, race and ethnicity, sex, health insurance, geographic location). We clustered SEs at the county level and reported adjusted odds ratios (AORs) with *P* values based on 2-tailed Wald χ^2^ tests derived from the logistic regression model, reporting results at a 5% significance level (*P* < .05). Given that airborne irritants, environment, and individual health conditions are the most common triggers of asthma,^33^ we expected sharp increases in the probability of asthma-related ED visits in the neighborhoods with the worst metrics of health and environmental conditions.^34^ To determine whether this was true, we used a piecewise linear logit model to estimate the patterns of health and environmental effects in neighborhoods. We estimated these effects on a subsample of patients, categorized by age, race and ethnicity, sex, and urban–rural residence. We estimated the percentage-point difference in predicted probabilities between low and very low opportunity neighborhoods, expressing this difference as the magnitude of the neighborhood effect specific to the very low opportunity level. We conducted all analyses in Stata version 18.0 (StataCorp LLC). The Texas A&M University Institutional Review Board determined this study to be exempt from oversight of research involving human subjects.

## Results

We identified 16 162 086 cases of ED visits, of which 343 886 (2.1%) were for asthma (eTable 1 in Supplement). The odds of an ED visit for asthma were highest in neighborhoods with very low COI across education, health and environment, and social and economic domains (Table). Health and environmental factors were the strongest predictors of asthma-related ED visits. Those living in neighborhoods with very low health and environmental opportunity were the most likely to visit the ED for asthma during the 4-year study period (AOR = 1.21; *P* < .001).

**Table.** Odds of visiting the emergency department for asthma among children and adolescents aged 2 to 17 years, by neighborhood Child Opportunity Index (COI) level, 9 US states, 2016-2019^a^

COI	Odds ratio (95% CI) [*P* value]				
	Very low	Low	Moderate	High	Very high
Education	1.09 (1.02-1.17) [.01]	1.05 (0.99-1.11) [.10]	1.04 (0.98-1.10) [.18]	1.02 (0.97-1.07) [.43]	1 [Reference]
Health and environment	1.21 (1.13-1.30) [<.001]	1.10 (1.03-1.18) [.01]	1.08 (1.01-1.14) [.01]	1.03 (0.99-1.08) [.18]	1 [Reference]
Social and economic	1.14 (1.07-1.22) [<.001]	1.05 (0.98-1.12) [.15]	1.03 (0.96-1.10) [.40]	1.02 (0.98-1.07) [.38]	1 [Reference]
Overall	1.14 (1.06-1.21) [<.001]	1.05 (0.98-1.12) [.15]	1.03 (0.98-1.10) [.32]	1.02 (0.97-1.06) [.38]	1 [Reference]

a The logistic regression results are displayed where opportunity levels were correlated with the binary independent variable indicating whether a child visited the emergency department for asthma (n = 16 162 085). Each row corresponds to the outcome from a separate regression model. Each regression model controlled for state, year, and correlates of individual characteristics. *P* values are based on 2-tailed Wald χ^2^ tests derived from the logistic regression model, with *P* < .05 considered significant. The 9 states were Arizona, Florida, Kentucky, Maryland, New Jersey, North Carolina, Oregon, Rhode Island, and Wisconsin.

**Figure 1.**
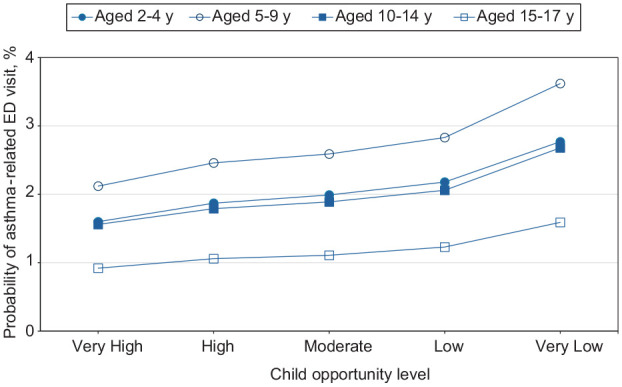
Estimated probability of asthma-related emergency department (ED) visits among children as a function of Child Opportunity Index levels (ie, a metric that describes and ranks neighborhood opportunity and the resources children need to grow up healthy, ordered from the lowest to the highest levels of opportunity US children experience), by group, in the 9 US states (Arizona, Florida, Kentucky, Maryland, New Jersey, North Carolina, Oregon, Rhode Island, and Wisconsin) studied during 2016-2019.

**Figure 2.**
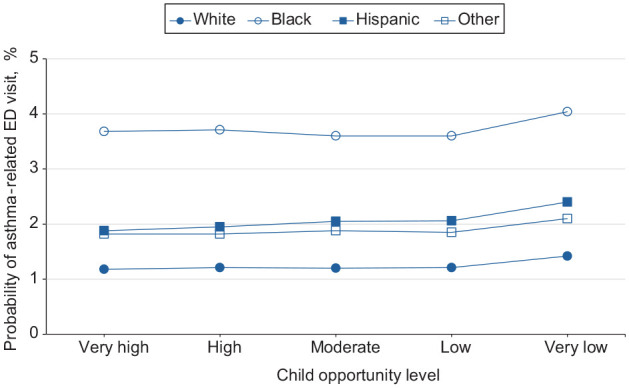
Estimated probability of asthma-related emergency department (ED) visits among children as a function of Child Opportunity Index levels (ie, a metric that describes and ranks neighborhood opportunity and the resources children need to grow up healthy, ordered from the lowest to highest levels of opportunity US children experience), by race and ethnicity, in the 9 US states (Arizona, Florida, Kentucky, Maryland, New Jersey, North Carolina, Oregon, Rhode Island, and Wisconsin) studied during 2016-2019.

**Figure 3.**
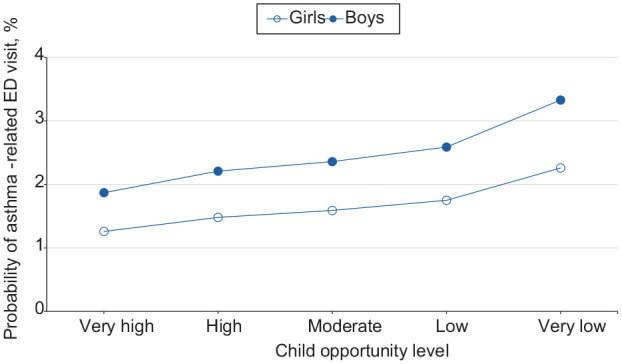
Estimated probability of asthma-related emergency department (ED) visits among children as a function of Child Opportunity Index levels (ie, a metric that describes and ranks neighborhood opportunity and the resources children need to grow up healthy, ordered from the lowest to highest levels of opportunity US children experience), by sex, in the 9 US states (Arizona, Florida, Kentucky, Maryland, New Jersey, North Carolina, Oregon, Rhode Island, and Wisconsin) studied during 2016-2019.

**Figure 4.**
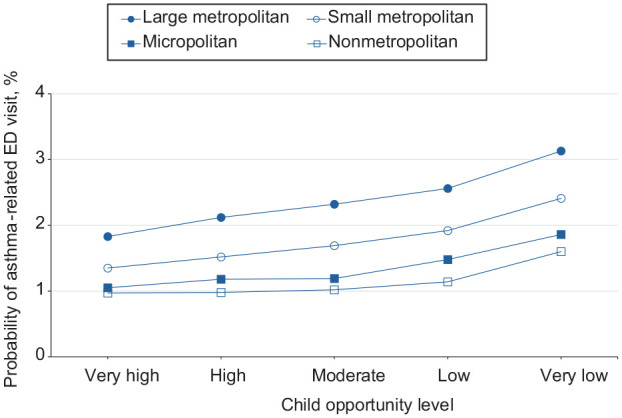
Estimated probability of asthma-related emergency department (ED) visits among children as a function of Child Opportunity Index levels (ie, a metric that describes and ranks neighborhood opportunity and the resources children need to grow up healthy, ordered from the lowest to highest levels of opportunity US children experience), by urban–rural residence, in the 9 US states (Arizona, Florida, Kentucky, Maryland, New Jersey, North Carolina, Oregon, Rhode Island, and Wisconsin) studied during 2016-2019.

We found the probability of ED visits across all patient groups increased as the level of neighborhood opportunity decreased, with the largest increase occurring when moving from a low to a very low opportunity level (eTable 2 in Supplement). We exhibited probabilities of asthma-related ED visits in the neighborhoods by age, race and ethnicity, sex, and urban–rural residence (Figures 1-4). The slope of the line connecting each pair of points reflected the size of the neighborhood effect in that portion of the neighborhood opportunity level. The slope between low opportunity and very low opportunity neighborhoods reflected the magnitude of the neighborhood effect at the very low end of the opportunity spectrum. For example, the estimated probability of ED visits for children aged 2 to 4 years increased from 2.2% to 2.8% as the level of neighborhood opportunity decreased from 2 (low) to 1 (very low) (Figure 1). This finding indicated that moving from a low opportunity neighborhood to a very low opportunity neighborhood corresponded to a 0.6 percentage-point increase in the probability of an asthma-related ED visit. For children aged 5 to 9, 10 to 14, and 15 to 17 years, moving from a low to a very low neighborhood opportunity corresponded to an 0.8, 0.6, and 0.4 percentage-point increase in the probability of an asthma-related ED visit, respectively (Figure 1). The probability of ED visits for asthma was highest among children aged 5 to 9 years at all opportunity levels. For children who were White, Black, Hispanic, and other races, moving from a low opportunity neighborhood to a very low opportunity neighborhood corresponded to a 0.2, 0.4, 0.3, and 0.3 percentage-point increase in the probability of an asthma-related ED visit, respectively (Figure 2). The effect of this shift for Black children was nearly twice that for White children. For all races, we found no neighborhood effect in the middle range of neighborhood opportunities. The probability of ED visits for asthma was highest among Black children at all opportunity levels.

For girls and boys, moving from a low to a very low neighborhood opportunity corresponded to a 0.5 percentage-point and 0.7 percentage-point increase in the probability of an asthma-related ED visit, respectively (Figure 3). The probability of ED visits for asthma was highest among boys at all opportunity levels. For children who resided in large metropolitan, small metropolitan, micropolitan, and nonmetropolitan/noncore areas, moving from a low to a very low neighborhood opportunity corresponded to an 0.6, 0.5, 0.4, and 0.5 percentage-point increase in the probability of an asthma-related ED visit, respectively (Figure 4). The probability of an ED visit for asthma was highest among residents of large metropolitan areas at all opportunity levels.

## Discussion

In this multistate, multiyear study, we found 343 886 children who had an ED visit for asthma. We correlated neighborhood opportunity levels with ED visits for asthma. Using adjusted logistic regression, we found that the odds of ED visits were highest in neighborhoods with the lowest level of opportunity. Furthermore, we found that neighborhood effects were largest in areas that scored lowest on health and environmental opportunities. In addition, we found that neighborhood effects were largest for children aged 5 to 9 years, Black children, boys, and those living in large metropolitan areas. These findings are consistent with studies that showed the odds of childhood asthma are high in neighborhoods with the lowest opportunity levels, and we argue that these high odds are mostly associated with health and environment as well as social and economic determinants of COI.^11,35,36^ Overall, our study uncovered a few important findings.

First, we found that movement from low to very low COI was associated with a sharp rise in the odds of visiting the ED for asthma. This increase might result from children’s extreme exposure to toxic elements or heat in neighborhoods with very low COI,^17^ given that both factors are known to contribute to an elevated risk of ED visits among children with asthma.^37,38^ In addition, children who reside in neighborhoods with very low COI may be exposed to poor housing quality.^17^ For these children, factors such as substandard housing that expose residents to allergens and lead may further elevate their risk of asthma-related ED visits.^39-41^ These findings align with previous research showing stark disparities in health outcomes between affluent and impoverished neighborhoods.^42^ Hence, the effect of very low COI on asthma-related ED visits could be mitigated by taking steps to transform neighborhoods with low COI into better resourced ones.^43,44^

Second, we found that the size of neighborhood effects varied depending on individual characteristics. For example, we found that neighborhood effects for Black children were nearly 3 times greater than those for White children. Moreover, we found that Black children across all COI levels (very low to very high) exhibited the highest likelihood of visiting an ED for asthma during the study period. While these findings are consistent with studies showing that race and ethnicity are independently associated with asthma-related ED visits,^39,45^ we are not aware of any causal studies that explain why large racial disparities in asthma-related ED visits persist, even when socioeconomic conditions are not adverse. However, several descriptive studies on the epidemiology of asthma suggest that these disparities may stem from financial and social hardships, educational disparities, or structural inequities that have historically contributed to large wealth gaps, even among families with similar incomes.^11,46^ Other studies have provided genetic explanations for some of the stark disparities in asthma outcomes among children.^47,48^ While these are all suggestive reasonings for observed disparities, we draw from studies in education^49^ and posit that long-term exposure to an area with limited resources may have lasting adverse effects on children’s health. Still, our conclusion is limited by the fact that we used claims data and did not have access to individual-level reports detailing wealth or health records. Future research should examine racial disparities at the upper levels of the COI.

Third, we found that the characteristics of children with a higher probability of ED visits for asthma mirrored those of children with a high prevalence of asthma at the national level. For example, the Centers for Disease Control and Prevention reported that in 2021, the prevalence of current asthma was highest among Black children, boys, and children older than age 5 years. We found that these same groups also had the highest likelihood of visiting an ED for asthma.^50^

Our study makes a unique contribution to the literature on neighborhood effects and asthma-related ED visits in several ways. First, by using discharge-level data, we were able to identify correlations between patient-level outcomes and neighborhood characteristics—an analysis not possible in studies using aggregated data. Second, we used rigorous evaluation methods to support the existence and significance of neighborhood effects on ED visits for asthma. We showed that neighborhood effects do not operate uniformly across subpopulations. Although we did not establish a causal relationship between the levels of opportunity in a child’s neighborhood and the likelihood of ED visits for asthma, our approach and large sample size allowed us to report the observed effects with greater confidence than in prior studies.

### Limitations

Our study had several limitations. First, we did not account for the selection of individuals into neighborhoods, which may have led to inflated estimates. This issue could have biased our results by producing negative effects for already disadvantaged populations who are concentrated in the same neighborhood and may be exposed to the spread of negative behaviors through peer influence. Second, while the COI is a validated metric and strengthened our study approach, it is a composite measure, making it difficult to isolate which component drives the observed effects and should therefore be targeted in interventions. In addition, our estimates may be biased because of unobserved confounders that influence pediatric asthma–related ED visits. Third, we were unable to distinguish and compare the composite effects of COI 2.0 indicators from their individual effects. As a result, we could not assess the relative value of analyzing the broader neighborhood context versus individual SDOH. Moreover, because of the nature of our data, we could not account for factors such as medication adherence, access to primary care, asthma severity, or outpatient asthma management. Lastly, we were unable to track individuals during the study period; therefore, we may have overlooked the effect of repeated ED visits on our estimates.

## Conclusions

Our findings highlight the need for targeted interventions to improve a neighborhood’s health and environmental resources and to promote health equity among demographic groups. Policies aimed at enhancing neighborhood resources in disadvantaged areas should be considered as a strategy to reduce asthma-related ED visits.

## Supplemental Material

sj-docx-1-phr-10.1177_00333549251361324 – Supplemental material for Exploring Neighborhood Opportunity as a Factor in Pediatric Asthma Visits to the Emergency Department Supplemental material, sj-docx-1-phr-10.1177_00333549251361324 for Exploring Neighborhood Opportunity as a Factor in Pediatric Asthma Visits to the Emergency Department by Nima Khodakarami, Marvellous Akinlotan and Alva O. Ferdinand in Public Health Reports®
