# Supplementary material for: Exploring Neighborhood Opportunity as a Factor in Pediatric Asthma Visits to the Emergency Department
Source: Public Health Rep. 2025 Aug 31;140(5-6):584–91. doi: 10.1177/00333549251361324 (PMC12399577; doi:10.1177/00333549251361324)
Supplement: Supplementary file 1 [file sj-docx-1-phr-10.1177_00333549251361324.docx]

Appendix

**Table 1** Demographic Characteristics of Children with Asthma Across the Child Neighborhood Opportunity Levels

|  | Overall | Nationally Normed Childhood Opportunity Index | | | | |
| --- | --- | --- | --- | --- | --- | --- |
|  |  | Very Low | Low | Moderate | High | Very High |
| **N Visits (%)** | 343,886 | 124,148 | 79,514 | 60,117 | 49,422 | 30,685 |
|  | (2.13) | (2.65) | (2.09) | (1.87) | (1.87) | (1.69) |
| **Age, N (%)** | | | | | | |
| 2-4 years | 90,459 | 33,505 | 20,654 | 15,128 | 12,983 | 8,189 |
|  | (2.20) | (2.59) | (2.06) | (1.84) | (1.97) | (1.95) |
| 5-9 years | 132,780 | 48,843 | 30,987 | 23,101 | 18,692 | 11,157 |
|  | (2.88) | (3.43) | (2.74) | (2.48) | (2.48) | (2.23) |
| 10-14 years | 83,591 | 29,545 | 19,359 | 14,956 | 12,139 | 7,592 |
|  | (2.07) | (2.60) | (2.02) | (1.80) | (1.75) | (1.51) |
| 15-17 years | 37,056 | 12,255 | 8,514 | 6,932 | 5,608 | 3,747 |
|  | (1.21) | (1.47) | (1.20) | (1.10) | (1.05) | (0.94) |
| **Race/ethnicity, N (%)** | | | | | | |
| White | 87,129 | 14,819 | 19,412 | 20,182 | 18,466 | 14,250 |
|  | (1.29) | (1.22) | (1.18) | (1.21) | (1.29) | (1.26) |
| Black | 152,555 | 74,326 | 34,506 | 21,234 | 15,036 | 7,453 |
|  | (3.91) | (4.05) | (3.62) | (3.46) | (3.51) | (3.46) |
| Hispanic | 85,979 | 30,035 | 21,542 | 15,315 | 12,729 | 6,358 |
|  | (2.16) | (2.19) | (2.17) | (2.02) | (2.08) | (1.93) |
| Other | 18,223 | 4,968 | 4,054 | 3,386 | 3,191 | 2,624 |
|  | (1.94) | (1.96) | (1.89) | (1.86) | (1.95) | (1.81) |
| **Sex, N (%)** | | | | | | |
| Female | 135,996 | 49,191 | 31,090 | 23,702 | 29,716 | 12,307 |
|  | (1.74) | (2.12) | (1.65) | (1.50) | (2.19) | (1.42) |
| Male | 207,890 | 74,957 | 48,424 | 36,415 | 19,706 | 18,378 |
|  | (2.59) | (3.18) | (2.53) | (2.23) | (1.53) | (1.92) |
| **Urban/Rural, N (%)** | | | | | | |
| Large Metro | 234,086 | 85,611 | 49,107 | 38,468 | 36,417 | 24,483 |
|  | (2.53) | (3.12) | (2.57) | (2.24) | (2.13) | (1.75) |
| Small Metro | 83,130 | 26,247 | 22,395 | 17,136 | 11,339 | 6,013 |
|  | (1.84) | (2.37) | (1.80) | (1.58) | (1.47) | (1.48) |
| Micro | 16,625 | 6,948 | 5,318 | 2,840 | 1,335 | 184 |
|  | (1.40) | (1.62) | (1.36) | (1.15) | (1.07) | (1.20) |
| Not metro | 10,045 | 5,342 | 2,694 | 1,673 | 331 | 5 |
|  | (1.17) | (1.31) | (1.07) | (0.97) | (0.97) | (0.25) |

**Table 2** Probability Estimates for Children of Different Characteristics Visiting ED for Asthma

| COI | Very Low | Low | Moderate | High | Very High  (ref group) |
| --- | --- | --- | --- | --- | --- |
| **Age, (SD)** | | | | | |
| 2-4 years | 2.77 | 2.18 | 1.99 | 1.87 | 1.60 |
|  | (1.31) | (1.15) | (1.07) | (1.06) | (0.84) |
| 5-9 years | 3.62 | 2.83 | 2.59 | 2.46 | 2.12 |
|  | (1.71) | (1.50) | (1.40) | (1.41) | (1.13) |
| 10-14 years | 2.68 | 2.06 | 1.89 | 1.79 | 1.56 |
|  | (1.32) | (1.14) | (1.06) | (1.05) | (0.86) |
| 15-17 years | 1.59 | 1.23 | 1.11 | 1.06 | 0.92 |
|  | (0.81) | (0.70) | (0.64) | (0.64) | (0.52) |
| **Race/ethnicity, (SD)** | | | | | |
| White | 1.42 | 1.21 | 1.20 | 1.21 | 1.18 |
|  | (0.56) | (0.47) | (0.46) | (0.46) | (0.47) |
| Black | 4.04 | 3.60 | 3.60 | 3.71 | 3.68 |
|  | (1.53) | (1.32) | (1.31) | (1.45) | (1.49) |
| Hispanic | 2.40 | 2.06 | 2.05 | 1.95 | 1.88 |
|  | (0.82) | (0.69) | (0.70) | (0.68) | (0.66) |
| Other | 2.10 | 1.85 | 1.88 | 1.82 | 1.82 |
|  | (0.77) | (0.66) | (0.70) | (0.68) | (0.72) |
| **Sex, (SD)** | | | | | |
| Female | 2.26 | 1.7 | 1.59 | 1.48 | 1.26 |
|  | (1.17) | (1.05) | (0.94) | (0.93) | (0.75) |
| Male | 3.33 | 2.59 | 2.36 | 2.21 | 1.87 |
|  | (1.66) | (1.44) | (1.34) | (1.34) | (1.07) |
| **Urban/Rural, (SD)** | | | | | |
| Large Metro | 3.13 | 2.56 | 2.32 | 2.12 | 1.83 |
|  | (1.60) | (1.40) | (1.29) | (1.33) | (1.09) |
| Small Metro | 2.41 | 1.92 | 1.69 | 1.52 | 1.35 |
|  | (1.23) | (1.08) | (0.99) | (0.86) | (0.71) |
| Micro | 1.86 | 1.48 | 1.19 | 1.18 | 1.05 |
|  | (1.04) | (0.92) | (0.66) | (0.62) | (0.46) |
| Not metro | 1.60 | 1.14 | 1.02 | 0.98 | 0.97 |
|  | (0.93) | (0.68) | (0.60) | (0.47) | (0.42) |

*Note*: The estimates from piecewise linear logit regression with standard deviations (SD) are presented here. All probabilities in the very low COI were statistically significantly higher than the referent group (p<0.001). COI levels are indicators of the Health and Environment.
